# TiO_2_ Coating and UV Photofunctionalization Enhance Blood Coagulation on Zirconia Surfaces

**DOI:** 10.1155/2019/8078230

**Published:** 2019-04-01

**Authors:** Khalil Shahramian, Aous Abdulmajeed, Ilkka Kangasniemi, Eva Söderling, Timo Närhi

**Affiliations:** ^1^Department of Prosthetic Dentistry and Stomatognathic Physiology, Institute of Dentistry, University of Turku, 20520 Turku, Finland; ^2^Turku Clinical Biomaterials Center (TCBC), University of Turku, 20520 Turku, Finland; ^3^Department of General Practice, School of Dentistry, Virginia Commonwealth University, Richmond, VA, USA; ^4^Clinic of Oral Diseases, Turku University Central Hospital, 20520 Turku, Finland

## Abstract

This in vitro study was designed to evaluate the effect of sol-gel derived TiO_2_ coating on blood coagulation, blood protein adsorption, and platelet response on zirconia surfaces. Square-shaped zirconia (n=96) (10x10x2 mm) was cut, ground, sintered, and finally cleansed ultrasonically in each of acetone and ethanol for 5 minutes. Three experimental groups (n=32) were fabricated: (a) zirconia coated with sol-gel derived TiO_2_, (b) zirconia coated with sol-gel derived TiO_2_ and treated with ultraviolet (UV) irradiation for 1 hour, and (c) non-coated zirconia as control. The coatings were prepared from tetraisopropyl orthotitanate solution by dip-coating. The thrombogenicity of the specimens was evaluated using a whole blood kinetic clotting time method where the extent of blood clotting was evaluated at 10, 20, 30, 40, 50, and 60 minutes (n=4/time point, total n=24/group). Scanning electron microscope images were taken to observe platelet morphologies after 1-hour incubation with platelet-rich plasma (PRP) (n=5/group). Surface characteristics were visualized using atomic force microscopy (n=1/group). Adsorption of plasma proteins and fibronectin on each surface was studied by gel electrophoresis (n=2/group). Significant differences were observed in blood coagulation between the test groups at 20-, 30-, 40-, and 50-minute time points (p<0.005). UV treated TiO_2_ coated specimens showed fastest blood coagulation followed by TiO_2_ coated and non-coated specimens. Furthermore, platelets appeared at a higher activation state on coated specimens. Gel electrophoresis revealed no difference in protein adsorption among the experimental groups. In summary, TiO_2_ coatings promoted blood coagulation, and it was further enhanced by UV treatment, which has the potential to hasten the wound healing process in vivo.

## 1. Introduction

Zirconia is being an increasingly used material in prosthetic dentistry [1]. The advantageous properties of zirconia as a biomaterial are a consequence of addition of yttria (Y_2_O_3_) to zirconia (ZrO_2_) crystals that yields what is commonly known as Yttria-stabilized Tetragonal Zirconia Polycrystal (Y-TZP) [2]. Once sintered, it has high bending strength and properties that are similar to stainless steel alloys [1, 2]. In addition to various applications, zirconia has also found its path into permucosal applications like implant abutments or copings for indirect restorations. When compared to titanium, zirconia abutments possess better light dynamics and hence, the final prosthesis is more esthetic to the human eye [1]. Moreover, it has been found to elicit less plaque accumulation and also provoke a weaker inflammatory response than titanium [1, 3, 4].

In the case of oral implants, one important success criterion is the bond and attachment between the implant abutment and the surrounding soft tissue [1, 5–10]. The formation of this permucosal seal protects the peri-implant environment by separating it from the intraoral environment, thereby blocking the passage of bacteria that can cause peri-implantitis [1, 10]. The formation of this soft tissue bond is highly dependent on the initial interactions of the abutment with blood [11, 12]. Blood is the first tissue that comes into contact with an implant or abutment surface at the surgical site; this is followed by adsorption of plasma proteins and blood cells on the surface [13–15]. Several authors have reported that the type of proteins adsorbed, hemostasis, and the formation of a blood clot are of decisive importance for the subsequent ingrowth of tissues and healing of the implants [11–13, 16]. Hemostasis involves an important step, which is blood platelets' activation. In addition, platelet activation releases cytokines and growth factors that play a prominent role during the subsequent peri-implant wound healing process [12]. Several studies have focused on developing surfaces that have a biological advantage to address these issues and to bond better with soft tissue [1, 17]. Sol-gel derived nanoporous titania (TiO_2_) coatings are one example of these surface treatments. Nanoporous TiO_2_ coatings are shown to improve soft tissue reaction with titanium and have also been deposited on different surfaces, including zirconia [9, 10, 17–19]. Furthermore, ultraviolet (UV) irradiation of TiO_2_ coatings has been shown to create an amphiphilic and a super-hydrophilic surface that is believed to be beneficial for its bioactivity [20]. UV irradiation can also be used as a chairside implant or abutment cleansing and sterilizing method to remove hydrocarbon contaminations or bacteria that are found to attach on the implants currently used in clinical or experimental settings [21–23]. However, astonishingly little research is available on the reaction of soft tissue with zirconia in general and also towards optimizing zirconia abutments for better soft tissue contact. This highlights the importance and the necessity of conducting more research in this field. Consequently, this study aims to explore the interactions of zirconia and sol-gel derived titania coated zirconia with blood, as an initial factor of the soft tissue zirconia interface. It is hypothesized that TiO_2_ sol-gel coating on zirconia will enhance blood coagulation, platelet activation, and plasma proteins adsorption. The present study is part of a series of studies attempting to improve the biological properties of zirconia implant abutments.

## 2. Materials and Methods

### 2.1. Specimen Preparation

A surgical saw (Struers Secotom-50, Copenhagen, Denmark) was used to cut a total of 96 square-shaped partially stabilized zirconia (METOXIT AG, Thayngen, Switzerland) at green stage (10x10x2 mm). Each specimen was then ground with silicon carbide paper (LaboPol 21, Struers A/S, Rodovre, Denmark) and sintered according to the manufacturer's instructions. The specimens were then put in baths of acetone and ethanol subsequently, where they were cleaned by ultrasound for 5 minutes. Three experimental surface treatments were made (n=32), (a) zirconia coated with sol-gel derived TiO_2_, (b) zirconia coated with sol-gel derived TiO_2_ and treated with ultraviolet (UV) irradiation for 1 hour, and (c) non-coated zirconia as control. In each experimental group, 24 of the samples were used for blood-clotting time measurement, 5 were used for platelet adhesion test, 2 were used for protein adsorption test, and 1 was used for surface analysis. The sol-gel solution was made by dissolving tetraisopropylorthotitanate [Ti(OCH(CH_3_)_2_)_4_] in 95% ethanol and mixing it with a solution of ethanol, nitric acid, and ultrapure water. The resultant solution was left to age at room temperature for 24 hours. The TiO_2_ coatings were prepared by dipping the specimens into the solution and then withdrawing them at a speed of 0.3 mm/s. Finally, the coated specimens were heated at 500°C for 1 hour and were again cleansed ultrasonically in each of acetone and ethanol for 5 minutes.

### 2.2. Blood-Clotting Time Measurement

Blood-clotting time was evaluated as previously described [12, 24, 25]. Human whole blood was drawn from a healthy nonsmoking female volunteer who had not taken any medications at least for the past 10 days. Since needle puncture causes the release of tissue thromboplastin, the first 3 ml of the blood was discarded. 100 *μ*l of the blood was then placed on the surface of each specimen (n=4/time point, total n=24/group) which was then put into 6-well plates and incubated at room temperature for 10, 20, 30, 40, 50, and 60 minutes. The wells containing the specimens were flooded with 3 ml of ultrapure water at the end of each time point. After 5 minutes, the resultant solution was sampled in triplicate (200 *μ*l each) and transferred to a 96-well plate. The addition of ultrapure water lyses any red blood cell that is not trapped in a thrombus, releasing their hemoglobin. An ELISA plate reader was used to measure the absorbance level of the solution at 570 nm, which correlates with the concentration of the released hemoglobin. The size of the clot formed is inversely proportional to the absorbance value.

### 2.3. Platelets Adhesion Test

Human whole blood was collected and mixed with 0.109 M solution of sodium citrate at a ratio of 9:1 (blood/sodium citrate solution). The platelet-rich plasma (PRP) was isolated by centrifugation of the mixture at 1500 rpm for 15 minutes at room temperature. Briefly, 100 *μ*l of the PRP was placed on the surfaces (n=5/group) of the specimens, which were then incubated for 1 hour at 37°C. Subsequently, the supernatant was discarded by rinsing the surfaces of the specimens thoroughly with phosphate buffered saline (PBS) for three times. Adherent platelets were then fixed for 2 hours at room temperature with 2.5% glutaraldehyde. All surfaces were then successively dehydrated at increasing alcohol concentrations (20%, 40%, 60%, 80%, 90%, and 100% for 15 minutes each). All dried specimens were mounted on a metal stub and were sputtered by 20 nm of gold using a sputter coater (Temcarb TB500, Emscope Laboratories Ltd., Ashford, United Kingdom). The surfaces of the specimens were then examined using a scanning electron microscope (SEM) (Leo Gemini 1530; Zeiss, Oberkochen, Germany) to count and observe platelet morphologies.

### 2.4. Plasma Protein Adsorption

Collection of adsorbed proteins was made with few modifications to the method previously described by Tanner et al. [26] All the specimens (n=2/group) were rolled for 30 minutes at room temperature in tubes containing human plasma that was diluted with PBS at a ratio of 1:4. The specimens were then washed twice with PBS. Each of the top and bottom surfaces of the specimens were rubbed with two microbrushes (Quick-Stick, Dentsolv AB, Saltsjö-Boo, Sweden) wetted with 4 *μ*l of sodium dodecyl sulphate polyacrylamide gel electrophoresis (SDS-PAGE) buffer (1 mM Na-phosphate buffer, 2% SDS, and 0.003% bromophenol blue) and finally with one dry microbrush. This desorbs the plasma proteins bound to the surfaces of the specimens. The tips of the microbrushes applied on each surface were then collected in an Eppendorf tube containing 20 *μ*l of the buffer, which was then heated in a boiling water bath for 7 minutes. The tubes were then perforated with a needle and placed in larger tubes that collected the sample solutions after centrifugation for 2 minutes (Heareus PICO17, ThermoFisher Scientific, Waltham, USA). Samples of duplicate specimens (from each surface) were collected in the same tube. The protein solutions were analyzed by SDS-PAGE and silver staining with the use of gradient Mini-Protean TGX gels (4-12%; Bio-Rad laboratories, Berkeley, USA). The resultant gels were observed and images were taken using an imaging system (ChemiDoc MP, Bio-Rad laboratories, Berkeley, USA). To evaluate the adsorption of fibronectin on the surfaces, the same procedure was repeated by rolling the specimens in a solution of 0.125 mg/ml bovine fibronectin (F4759 Sigma, Sigma-Aldrich, St. Louis, USA).

### 2.5. Atomic Force Microscopy (AFM)

Surfaces (n=1/group) were imaged with atomic force microscope (AFM) (NNTEGRA Prima, NT-MDT, Russia). Images of size 5 *μ*m by 5 *μ*m were taken with a resolution of 1024 x 1024 pixels using a HQ:NSC14/Al BS cantilever (*μ*masch, Estonia) (T=24+/-1 C, RH%=37.5+/-2.5). The software Scanning Probe Image Processor (SPIP, Image Metrology, Denmark) was used to analyze the height profiles.

### 2.6. Statistical Analysis

Statistical analysis was performed with Statistical Package for the Social Sciences (Version 23.0, SPSS, Chicago, IL). Repeated measures analysis of variance (ANOVA) was used as the statistical test for the comparison of several means. Pairwise comparisons among the means were made using simple contrasts with Bonferroni corrected p-values.

## 3. Results

**Figure 1:**
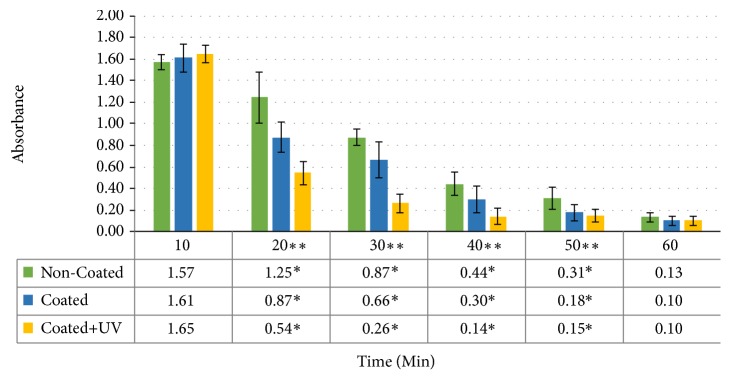
Blood-clotting profiles for non-coated, TiO_2_ coated, and UV irradiated TiO_2_ coated zirconia. The plot shows the optical density versus time. Data are presented as mean ± standard deviation (n=4/time point). Table below the chart represents the respective absorbance values at each time point.  ^*∗*^Statistically significant differences between the groups at the marked time points (p<0.005).  ^*∗∗*^Statistically significant differences between the time points (p<0.001).

Blood-clotting profiles for the three surface treatment groups are shown in Figure 1. Both the test groups, i.e., TiO_2_ coated, in addition to the ones treated with UV irradiation, showed faster blood coagulation. Significant differences were already observed at the 20 minute time point where both test groups demonstrated lower absorbance values compared to the control group, reflecting higher amount of blood clotting (p<0.005). Similarly, the same differences were also present at the 30-, 40-, and 50-minute time points. Between the test groups, TiO_2_ coated+UV treated specimens represented the highest extent of blood clotting at all the forenamed time points (p<0.005). Blood is considered to have clotted completely when the optical densities drop below 0.3. The time taken for blood coagulation, in other words the total clotting time, was almost 30 minutes for the TiO_2_ coated+UV treated specimens followed by 40 minutes and 50 minutes for the TiO_2_ coated and non-coated control specimens, respectively. Moreover, statistically significant differences were also observed among the time points, stating that blood coagulation was more profound with the passage of time (p<0.001) (Figure 1).

**Figure 2:**
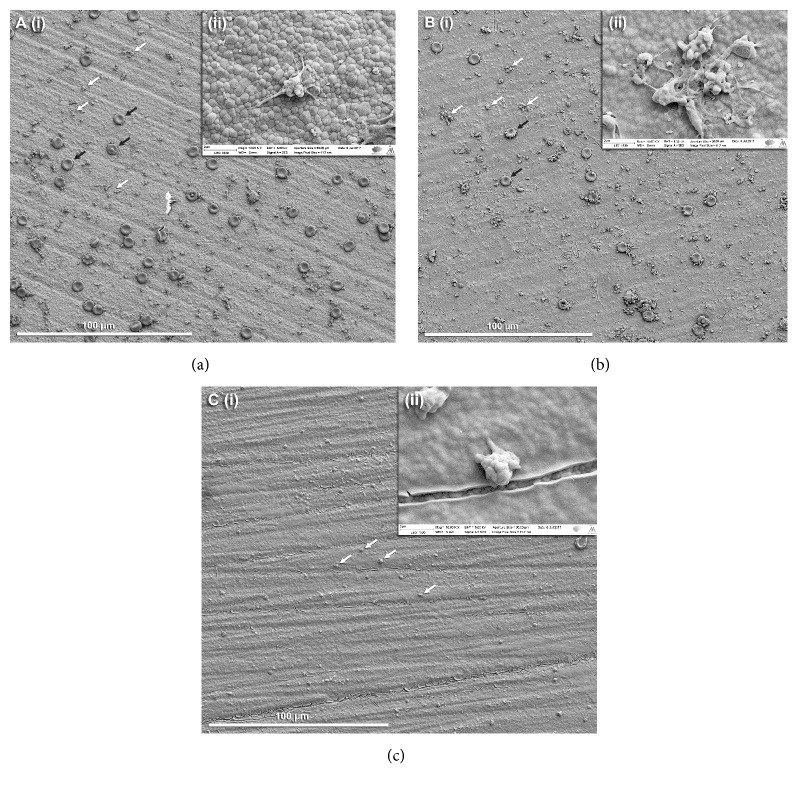
Scanning electron micrographs of platelet morphologies after 1-hour adhesion period on (i) panogramic image of 30 stitched images of the surface wetted with platelet-rich plasma (PRP) at x2500 magnification. (ii) x10000 magnification of platelet morphologies. (a) Non-coated zirconia, (b) zirconia coated with TiO_2_, and (c) zirconia coated with TiO_2_ irradiated with ultraviolet radiation.* Note*. White arrows show platelets; black arrows show red blood cells.

Figure 2 shows the platelet morphology after the 1-hour adhesion period. Panoramic images (Figure 2(i)) represent 30 stitched images of the surface taken at x2500 magnification. All groups showed platelet adhesion; however, differences were observed in the morphologies of the adhered platelets. The platelets on the TiO_2_ coated specimens were more spread, more dendritic, more aggregated, and therefore more active compared to the ones observed on the control, non-coated specimens. On the other hand, the platelets on the surface of TiO_2_ coated+UV treated specimens were discoid or round, which translates to a lower state of activation.

**Figure 3:**
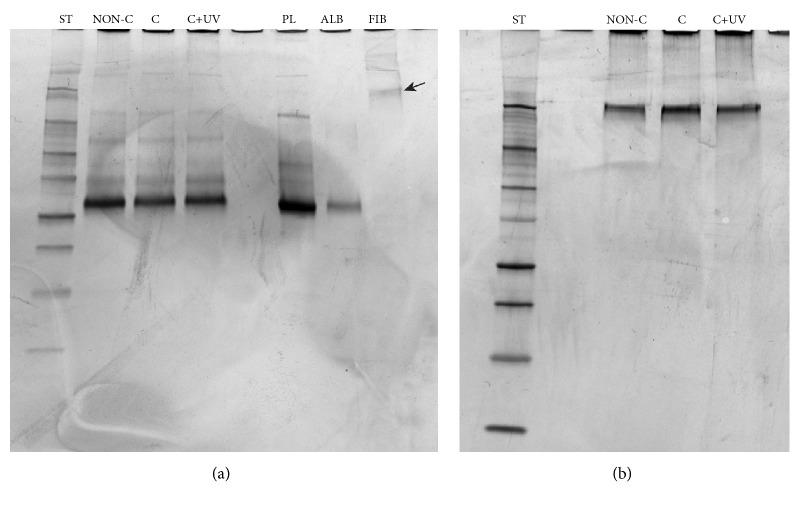
Gel electrophoresis for (a) plasma proteins and (b) fibronectin adsorbed on the different test materials; NON-C: non-coated zirconia, C: zirconia coated with TiO_2_, and UV: zirconia coated with TiO_2_ irradiated with ultraviolet radiation. Black arrow in Figure 3(a) represents the band identifying fibrinogen. The protein standard contained proteins with the following molecular weights, in kD: 250, 150, 100, 75, 50, 37, 25, 20, 15, and 10. Plasma: PL; albumin: ALB; fibrinogen: FIB.

All the surfaces adsorbed a broad range of plasma proteins and no consistent differences in the protein profiles were noticed. Albumin was the protein showing the highest adsorption. The amount of fibronectin adsorbed on the surfaces seemed also to be on the same level in all the three groups (Figure 3).

**Figure 4:**
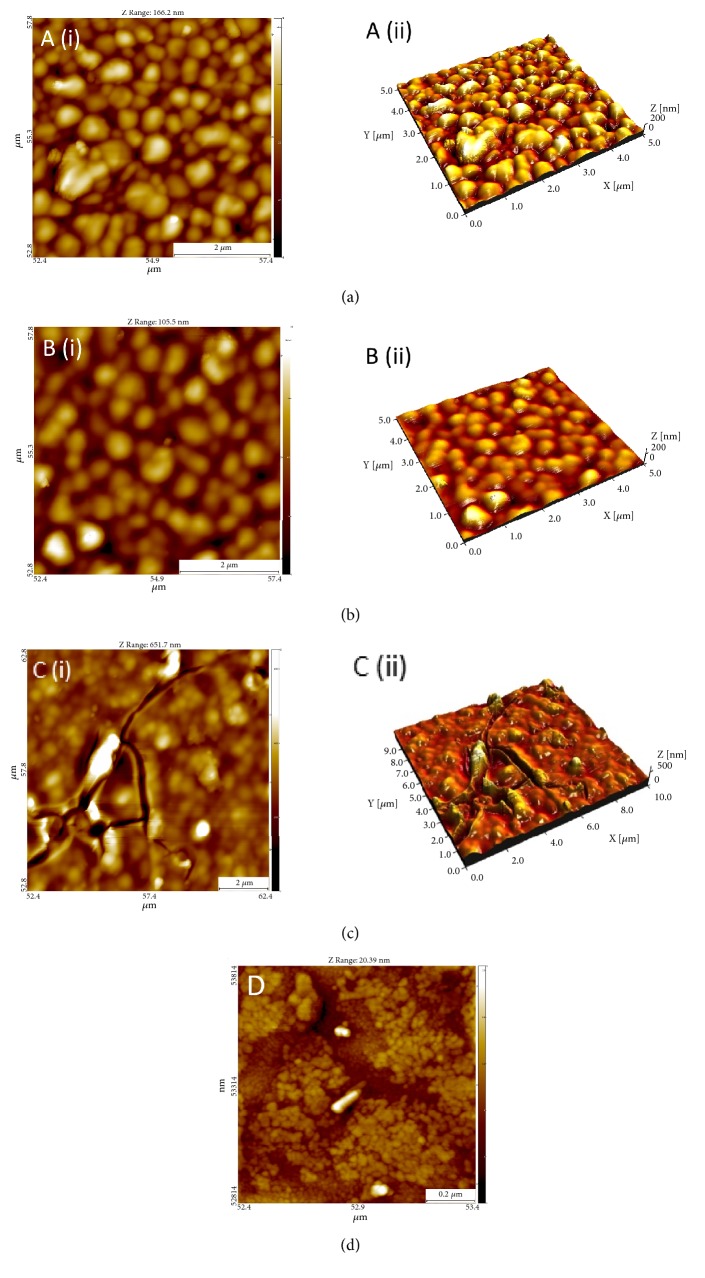
Atomic force microscope images (AFM) of (a) non-coated zirconia (b) and (c) TiO_2_ coated zirconia. (i) Two-dimensional (2D) AFM image. (ii) Three-dimensional (3D) AFM image. (d) Two-dimensional (2D) AFM image revealing the nanotopography of TiO_2_ coated zirconia.

On both the surfaces, granular nanostructures ranging from a few ten to several hundred nanometers could be seen. The smoothening effect of sol-gel coating on zirconia surface can be seen in the AFM images which are taken on a microscale, 5 *μ*m x 5 *μ*m and 10 *μ*m x 10 *μ*m (Figure 4(a) versus Figure 4(b)).  Figure 4(c) identifies presence of cracks within the coating, and clearly shows the smoothening effect of sol-gel derived TiO_2_ coatings on the surface of zirconia. However, the TiO_2_ coatings alter the nanotopography of the zirconia surface and the resultant surface is more rough than the non-coated controls (Sa 25.8 nm and Sa 19.4 nm, respectively). The nanotopography and the granular surface of the TiO_2_ coating can be observed in Figure 4(d).

## 4. Discussion

This study showed that sol-gel derived TiO_2_ coatings and subsequent UV light treatment enhanced blood coagulation on zirconia surface. Zirconia is widely used as an abutment material replacing conventional titanium metal where there is a demand for better esthetics and in patients with metal allergies [27–30]. However, in order to achieve a long-term stability of a satisfactory implant treatment, it is important that the health of the soft tissue around implants can be maintained. Formation of a healthy gingival attachment is highly dependent on the uneventful healing of the tissues after the abutment surgery. The healing of the soft tissue starts immediately during the surgery by protein adsorption on implant or abutment surface, followed by the formation of a blood clot at the site of surgical wound. The wound is rapidly sealed off by the blood clot and later on, this fibrin clot induces an inflammatory process that leads to tissue formation and remodeling [31–35]. The formation of blood clot is crucial because it serves as a pathway for the migration of cells to reach the implant surface. In other words, the cells do not interact directly with the material surface, but with a blood modified material surface instead [13, 36].

The potential biological benefits of sol-gel derived TiO_2_ coatings on zirconia have been previously demonstrated by the authors [10]. TiO_2_ coatings improved fibroblast proliferation and the coating procedure did not affect the favorable mechanical properties of zirconia. Further steps were taken in this study to test the thrombogenicity of TiO_2_ coatings and their UV photofunctionalization. Whole blood was used to mimic a clinical blood/abutment interaction under a static and air-contacting condition. Clinically, implants or abutments come into contact with a pool of blood at the surgical site that is exposed to air. Therefore, the abutment surface, air, and blood all contribute to the abutment/blood interactions and the interface created. Furthermore, the use of anticoagulated blood is discouraged by Johnson et al. [37] due to the effects that anticoagulation substances have on the biological behaviors of blood. Use of blood derived from a single donor is a limitation of this study in order to draw general conclusions for the public. However, this study aimed at looking at the behavior of different surfaces with blood, and the behavior of different blood types from different individuals is out of the scope of this study. Interindividual variations in blood coagulation might have resulted in different total coagulation times, but it is unlikely that increasing the number of donors would have affected the coagulation profiles among the experimental material groups. Furthermore, Tan et al. recently demonstrated that blood properties including blood coagulation mediators and time are independent of race and racial differences [38]. The optical density (OD) of the hemolyzed blood solution decreases with time (Figure 1). A lower OD value means a lower hemoglobin concentration in whole blood solution, which translates to a more extensive thrombus formation on the specimens. The results of this study clearly indicated that TiO_2_ coatings have better blood-clotting ability than plain zirconia. This ability was further enhanced with UV irradiation of the specimens prior to the start of the experiment, confirming the first hypothesis, while contradicting the results of previous research on the effects of UV treatment on blood coagulation [23, 39, 40]. SEM images (Figure 2) were obtained to study the adhered blood platelets on the specimens. Platelet activation is a crucial event in the formation of a thrombus, and it involves a series of morphological changes and cytoskeletal rearrangements, in addition to release of chemicals involved in the wound healing process. Platelets on the surface of coated specimens were at a higher state of activation (early pseudopodial) compared to the other groups. The effect of inhibition of platelet adhesion by UV irradiation has been demonstrated in previous studies [23, 39, 40]. Goodman et al. [41] have classified the steps of platelet activation into five categories: (a) discoid or round; (b) dendritic; (c) early pseudopodial, spread dendritic; (d) intermediate pseudopodial, spreading; and (e) fully spread. Hence, the adherent platelets on plain zirconia (dendritic) and UV treated specimens (discoid) were in a lower activated state compared to the ones on coated specimens (spread dendritic), which supports the acceptance of the second hypothesis that TiO_2_ coatings improve thrombogenicity of zirconia.

The study of protein adsorption on the surface of a newly developed biomaterial is of utmost importance [42, 43]. The first major event induced when the biomaterial comes into blood contact is the adsorption of proteins on its surface [25, 44, 45]. It has been stated that the initial layer of adsorbed albumin will be replaced by adhesion proteins that are recognized by integrin receptors. This transforms the biomaterial to become suitable for adhesion of cells and consequent integration of tissue [42, 43]. Fibronectin is an extracellular matrix protein known to play a role in cell attachment, spreading, and differentiation in addition to platelet adhesion and aggregation [46]. Several studies have elucidated the adsorption of plasma proteins on different surfaces; however, most of these experiments are limited to the adsorption of one or few proteins [47–50]. Different approaches have been taken towards the identification of these proteins and SDS-PAGE was chosen in this study similarly to its previous use to investigate protein adsorption on bioactive glasses, where differences were successfully identified [44, 49, 51–53]. Different plasma proteins (predominantly albumin) and fibronectin were adsorbed on the surfaces of all three tested groups, although no consistent differences were observed among them (Figure 3). The literature supports that rough implant surfaces promote blood coagulation and the process of osseointegration [54, 55]. However, it has been demonstrated that fibroblasts prefer smooth surfaces and that a surface roughness of 0.2 *μ*m is considered to be a threshold for soft tissue attachment [56]. Above this threshold, bacterial biofilms colonize the surface more than cells and tissue. The coating employed in this study is made of nanoparticles of anatase TiO_2._ The nanoscale roughness of the coated zirconia grains is higher than non-coated zirconia grains. In a recent study employing human dermal fibroblasts, Rosqvist et al. found that surfaces with roughness of approximately Sa 11 nm favored the proliferation yield of cells [57]. The study also concluded that surface effects on cell proliferation stems from nanoscale roughness [57]. The surface analysis through AFM in our study supports the findings of previous studies. In the present study, zirconia coated with TiO_2_ is found to be rougher (Sa 25.8 nm) compared to non-coated controls (Sa 19.4 nm) on a nanoscale level. It can be suggested that the faster blood coagulation observed on the coated samples may also be a result of the higher nanoscale roughness. The coated zirconia is also within the roughness threshold for soft tissue attachment, and as a result, is therefore hypothesized to have a faster wound healing and hence better attachment to the surrounding tissue. Nevertheless, the results reported highlight the great importance of nanotopography and nanoscale roughness when studying the surface characteristics of biomaterials.

It has been reported that UV treatment induces a positive surface charge on the surface of TiO_2_ and hence promotes the adsorption of proteins and adhesion of cells [22, 57, 58]. On the other hand, some papers also report that it prevents fibrinogen and platelet adhesion and therefore conclude that UV irradiation of a TiO_2_ coated surface enhances its anticoagulant properties [23, 39, 40]. The data presented currently in the literature regarding the effects of UV irradiation on TiO_2_ surfaces is conflicting [39]. The anticoagulative reports made by these previous papers are based on extrapolating results obtained from experiments conducted solely with synthetic blood proteins and blood platelets. Fresh whole blood used in this study is more justified to draw more reliable conclusions. The coagulation cascade is a complicated process involving several reactions including the intrinsic pathways, extrinsic pathways, and common pathways [59]. Influence on any of these several reactions can positively or negatively affect blood coagulation. More research has to focus on addressing the conflicting reports of the literature on effect of UV treatment on blood coagulation. Furthermore, this study is the first to test zirconia, which is being increasingly used as a dental implant abutment material. These factors all highlight the importance of future focus into this topic. Nevertheless, although essential, all these experiments are a fraction of the very many events happening at the site of abutment contact with soft tissue in a living oral environment, and other intraoral factors must be also put in consideration.

## 5. Conclusion

Within the limits of this study it can be concluded that TiO_2_ coatings promote blood coagulation, a property that is further enhanced by UV treatment. Furthermore, TiO_2_ coatings alone also promoted platelet adhesion and activation. Enhanced blood coagulation results in faster wound healing process that can be helpful in final attachment of the abutment to the surrounding soft tissue.

## Data Availability

The authors declare that the data supporting the findings of this study are available within the paper. In case of further requirements, the corresponding author can be contacted for assistance.
